# Electrical and Immunohistochemical Properties of Cochlear Fibrocytes in 3D Cell Culture and in the Excised Spiral Ligament of Mice

**DOI:** 10.1007/s10162-021-00833-z

**Published:** 2022-01-18

**Authors:** A. Osborn, D. Caruana, D. N. Furness, M. G. Evans

**Affiliations:** 1 School of Life Sciences, Keele University, Stoke-on-Trent, ST5 5BG UK; 2 Present Address: Life & Health Sciences, Aston University, Birmingham, B4 7ET UK

**Keywords:** Cochlea, Fibrocyte, Collagen hydrogel, Immunolabelling, Whole-cell recording

## Abstract

Fibrocyte degeneration in the cochlear lateral wall is one possible pathology of age-related metabolic hearing loss (presbycusis). Within the lateral wall fibrocytes play a role in potassium recycling and maintenance of the endocochlear potential. It has been proposed that cell replacement therapy could prevent fibrocyte degeneration in the CD/1 mouse model of hearing loss. For this to work, the replacement fibrocytes would need to take over the structural and physiological role of those lost. We have grown lateral wall fibrocytes from neonatal CD/1 mice in a 3D-collagen gel culture with the aim of assessing their functional similarity to native lateral wall fibrocytes, the latter in a slice preparation and in excised spiral ligament pieces. We have compared cultured and native fibrocytes using both immuno-labelling of characteristic proteins and single cell electrophysiology. Cultured fibrocytes exhibited rounded cell bodies with extending processes. They labelled with marker antibodies targeting aquaporin 1 and calcium-binding protein S-100, precluding an unambiguous identification of fibrocyte type. In whole-cell voltage clamp, both native and cultured fibrocytes exhibited non-specific currents and voltage-dependent K^+^ currents. The non-specific currents from gel-cultured and excised spiral ligament fibrocytes were partially and reversibly blocked by external TEA (10 mM). The TEA-sensitive current had a mean reversal potential of + 26 mV, suggesting a permeability sequence of Na^+^  > K^+^. These findings indicate that 3D-cultured fibrocytes share a number of characteristics with native spiral ligament fibrocytes and thus might represent a suitable population for transplantation therapy aimed at treating age-related hearing loss.

## INTRODUCTION

Hearing impairments are one of the biggest health-related problems reported worldwide. The World Health Organisation estimates that over 5% of the world’s population have debilitating hearing loss. Presbycusis is a form of hearing loss associated with normal ageing, with around 43% of elderly individuals affected (age range 65–84, Nash et al. 2011). Typically, the patient audiogram shows a monotonic decrease in hearing sensitivity with increasing frequency. However, recent quantitative analyses of human audiometric data suggest that there are other patterns of presbycusis (Dubno et al. 2013). Thus, presbycusis can relate to sensory cell and neuronal losses (defined as sensorineural hearing loss) and cochlear lateral wall pathology (defined as metabolic hearing loss). The proportion of these different types is, as yet, still to be fully determined but from the data in Dubno et al. (2013) and Schuknecht and Gacek (1993); metabolic hearing loss is at least as prevalent as sensorineural, and may even be more prevalent, although Wu et al. (2020) suggest that hair cell loss is the predominant factor.

In animal models, loss of fibrocytes from the cochlear lateral wall appears to precede hair cell and neuronal loss in mouse models which show presbycusis (Hequembourg and Liberman 2001; Mahendrasingam et al. 2011b), and thus their replacement might be expected to improve hearing in these cases. Fibrocytes remain a promising focus for regeneration therapy. For example, following noise-induced hearing loss in mice, hearing recovery has been linked to the natural repopulation of fibrocytes within the lateral wall, apparently caused by type III fibrocytes migrating from their usual inferior position into the region bordering the *stria vascularis* where type I fibrocytes reside and where cells were lost (Li et al. 2017). Type III fibrocytes appear to possess a proliferative capacity that aids repair of the lateral wall following noise damage (Li et al. 2017).

The cochlear lateral wall comprises the spiral ligament together with the medially located *stria vascularis*. Together they are essential for the generation of the positive endocochlear potential, generated by a process of transcellular K^+^ movement from the organ of Corti to the *stria vascularis* via the syncytium of the fibrocytes in the lateral wall (Adachi et al. 2013; Furness 2019). Five fibrocyte types have been described in the lateral wall (Spicer and Schulte 1996), each within a distinct region with a distinctive morphology and having differentially distributed marker proteins including K^+^ transporting proteins, such as the Na^+^ K^+^ ATPase pump (Schulte and Adams 1989), the Na^+^K^+^2Cl^−^ cotransporter (Crouch et al. 1997) and the Kir 5.1 channel (Hibino et al. 2004).

Cochlear fibrocytes can be successfully cultured in vitro enabling the exploration of protein expression and ion channel physiology (Liang et al. 2004, 2005; Shen et al. 2004). The most common fibrocyte type to be identified in culture is type I (Gratton et al. 1996; Suko et al. 2000; Liang et al. 2003; Shen et al. 2004), although cultures of type III (Kelly et al. 2012) and type IV (Qu et al. 2007) fibrocytes have also been established. In vivo microelectrode measurements have indicated that the fibrocyte syncytium, established by gap junctions between type I fibrocytes and the basal cells of the stria vascularis, and between type I fibrocytes and adjoining type II and type V fibrocytes, has a positive membrane potential (0 to + 7 mV relative to the perilymph), due to a large cell membrane Na^+^ permeability (Yoshida et al. 2016). This contrasts with whole-cell recording measurements in cultures and in slices where negative membrane potentials were directly measured (Shen et al. 2004) or inferred from the zero-current potential in whole-cell voltage-clamp (Furness et al. 2009). These very different experimental approaches, coupled with other differences, such as age, developmental stage, species and cochlear location, preclude definitive conclusions at present.

We have grown cochlear fibrocytes on collagen I gels in order to assess their electrical properties and their labelling for key fibrocyte marker proteins. We chose to culture the cells on collagen hydrogels, rather than on flat surfaces, since this approach produces fibrocytes exhibiting a more natural morphology (Mahendrasingam et al. 2022). We also compared their electrical properties with those of fibrocytes within the lateral wall, either in dissected portions or in cochlear slices. Given that a fibrocyte-replacement therapy might be a useful tool to combat presbycusis, the aim of this study was to assess the extent to which fibrocytes grown in 3D cell culture have the same functional characteristics as native lateral wall fibrocytes.

## MATERIALS AND METHODS

### Tissue Preparation and Cell Culture

CD/1 mice were bred and maintained in our Central Animal Facility. All the animals were treated in accordance with the UK Animal (Scientific Procedures) Act of 1986, and their use was approved by the Animal Welfare and Ethical Review Board at Keele University. Mice pups (P6-P10) were killed by cervical dislocation and then decapitated following UK Home Office schedule 1 guidelines. The cochlea was dissected from the skull and placed in cold culture medium (4 °C) and examined using a dissecting microscope. Sterile forceps were used to chip away the outer bone to reveal the inner spiral. Lengths of the lateral wall were detached and the *stria vascularis* was removed leaving the spiral ligament. The spiral ligament was divided into shorter pieces to be used in cell cultures or for patch clamp recording; all regions were used. For the cultures, the spiral ligament tissue fragments were placed, strial side down, into 6-well plates filled with 50 µl of culture medium (MEM-α supplemented with 10% FCS (fetal calf serum), 1 × PSF (penicillin–streptomycin-fungizone) and 1% ITS-G supplement (Thermo Fisher Scientific: bovine insulin, human transferrin and sodium selenite)) and covered with a glass cover slip. The well plates containing the ligament were placed in an incubator at 37 °C (5% CO_2_, 95% O_2_) for 24 h. The cover slip was removed and 2 ml of culture medium was slowly added before placing back into the incubator. Culture medium was changed every 2 to 3 days after rinsing twice with the washing buffer (sterile PBS with antimycotic antibiotic). For patch clamp recording, the spiral ligament tissue fragments were secured (usually bone-facing side down) in the recording chamber filled with external solution (see below) under two pieces of teased dental floss arranged at right angles. During the dissection, a cut was made in the tissue to denote the direction of the apex and the superior edge. Most of the successful recordings were made in the type II/IV region of the spiral ligament.

### Preparation of Collagen I Gels

Collagen I gels (3 mg in 1-ml DMEM) were prepared following manufacturer’s guidelines (Thermo Fisher Scientific UK) and seeded with cells after about 1 h. All the solutions were kept on ice. A volume of 250 µl of the gel was placed on the centre of a 30-mm sterile petri dish, with the gels having a diameter of ~ 16 mm and incubated at 37 °C for at least 30 min. Cells were seeded directly on top of the gels using 50 µl of cell suspension containing a density of approximately 5 × 10^4^ cells per sample and left to settle for ~ 2 h after which fresh culture medium (MEM-α supplemented as indicated above) was gently added to cover them overnight. For immunocytochemistry, gel cultures were fixed in 4% paraformaldehyde for 2 h after which they were stored in 0.4% paraformaldehyde.

### Preparation of Cochlea Slices

Cochlear slices were taken from P7-9 CD/1 mice, an optimal age for slicing of the cochlea (Jagger et al. 2000). The bullae were excised and the cochlear bones exposed. Additional dissection was performed in 4 °C artificial cerebrospinal fluid (ACSF), containing (mM) NaCl 124, KCl 3, CaCl_2_ 3, MgCl_2_ 3, NaH_2_PO_4_ 1.3, glucose 10, HEPES 10 and pH 7.4. The cochlea was mounted on a vibratome (Leica VT1000) using superglue, and slices were cut horizontally from apical to basal end in a mid-modiolar plane at 300 µm using a vibrating blade (8 Hz) at 1.5 mm/s. The slices were then submerged in the cold ACSF and transferred into a recording chamber with the spiral ligament exposed to allow pipette access to the fibrocytes. Most of the recordings were obtained in the inferior part of the spiral ligament, where fibrocyte types II and IV are found.

### Immunocytochemical Labelling

For immunofluorescent labelling of cultured and native cells, samples were fixed in 4% paraformaldehyde for 2 h as described above, washed three times with a phosphate buffer and once with phosphate buffer saline (PBS) before following similar immunolabelling procedures used previously (Mahendrasingam et al. 2011a). In brief, they were permeabilised with 0.25% Triton-X-100 in PBS with 1% goat-serum for 30 min. The gel was then washed with PBS × 3 and pre-blocked with 10% goat-serum PBS for 30 min. Samples were then incubated overnight at 4 °C on a rotator with AQP1 at 1:100 (rabbit, AB3272-50UL, Merck, UK) and S-100 at 1:100 (goat, sc-7851, Santa Cruz, USA). The following day the gel was washed with 1% goat-serum PBS × 3 followed by application of the appropriate conjugated secondary antibodies (AlexaFluor 488 green and 568 red) for 2 h at room temperature. After this, the gels were washed in PBS × 3, mounted in antifade solution and observed on a confocal microscope (MRC 1024, BioRad, UK). The antifade solution comprised 0.01% p-phenyldiamine (w/v) and 1.5% polyvinyl alcohol (w/v) in glycerol/0.1-M phosphate buffer (3:7 v/v).

### Confocal Fluorescence Microscopy Imaging

The confocal microscope (MRC 1024) was equipped with a krypton-argon laser with excitation lines at 488 nm and 530 nm. Fluorescence was detected using in-line filters for 510–530 nm (green, 488-nm excitation) and 565–600 nm (red, 530-nm excitation). Three individual fluorescent images over the full z-axis range were captured with Lasersharp2000 software and merged into a single representative image using Adobe Photoshop (version 7.0).

### Whole Cell Patch Clamp Recording

The recording chamber was continuously perfused with an extracellular solution containing (mM) NaCl 124, KCl 2.5, CaCl_2_ 1.3, MgCl_2_ 2, NaH_2_PO_4_ 1.4, glucose 17, NaHCO_3_ 26 (5% CO_2_/95% O_2_) and pH 7.4. The intracellular solution contained (mM) KCl 140, NaCl 10, MgCl_2_ 2, EGTA 0.5, glucose 5, HEPES 5, and pH 7.3. In some experiments, a fluorescent dye (0.1-mM dextran 3000, Thermo Fisher Scientific, UK) was added to the intracellular solution to aid cell identification. The chamber was mounted on the stage of an Olympus BX microscope equipped with × 4 air and × 40 water-immersion objectives, a blue LED for epifluorescent illumination, a fluorescein filter set and a CMOS camera (optiMOS, QImaging).

The patch electrode with positive back pressure (resistance 3MΩ) was directed towards the cell body using a micromanipulator (Scientifica, UK) until the tip touched the cell, and a small dimple was observed. A high resistance seal was obtained following release of the pressure with application of negative pressure by mouth and the holding potential was set to -50 mV. Following break-in, voltage steps were delivered to activate the membrane currents (see Figs. 4, 6 and 7). Initially, a small voltage step (10 mV) was delivered in order to compensate, as much as possible, the cell capacitative transient using the amplifier controls (Heka, EPC-7). In cells where capacitance was measured, it was read directly from the amplifier slow capacitance potentiometer. Recordings were acquired and analysed using Signal 2.16 software and a micro-1401 interface (CED, UK). Voltages were not corrected for series resistance (typically 6MΩ) or junction potential (calculated to be − 3 mV). Membrane potential was measured as the zero-current voltage from the current–voltage plots in voltage clamp. In most cases, voltage increments were set at 10 mV or occasionally 20 mV (see Fig. 4). TEA (10 mM) dissolved in extracellular solution was pressure-ejected from a second patch pipette positioned 10–20 µm from the cell (pneumatic picopump, PV820, WPI, USA). A two-step voltage clamp protocol was used to investigate the blocking actions of TEA. The steps were 100-ms long and voltage increments were equal. The TEA application was synchronised with each triggered acquisition so that the second of the steps was delivered towards the end of a 0.6-s application of TEA. A few seconds gap between each pair of steps allowed sufficient time for baseline recovery, the first step of each pair acting as a control. It was important to obtain a complete family of responses in the shortest time, thus allowing both a stable comparison between control and test (TEA) for all the steps and an estimate of the reversal potential. In these experiments, the membrane potential was also inferred from zero current voltage, although due to differences in the voltage-clamp protocols, these data were not included in the comparative membrane potential data (Fig. 5A).

### Statistics

Data were analysed using GraphPad Prism software (versions 8,9). Data are given as mean ± standard deviation (SD). Tests for normal distribution and equal variance were conducted to determine a suitable parametric or non-parametric test. Significance was assessed at the 5% level (*p* < 0.05). In order to minimise series resistance errors and to avoid sampling from overly leaky cells, cells with a holding current of > 0.7 nA at − 50 mV were excluded from the analysis of fibrocyte groups.

## RESULTS

### Cultured Spiral Ligament Fibrocyte Characterisation

**Fig. 1:**
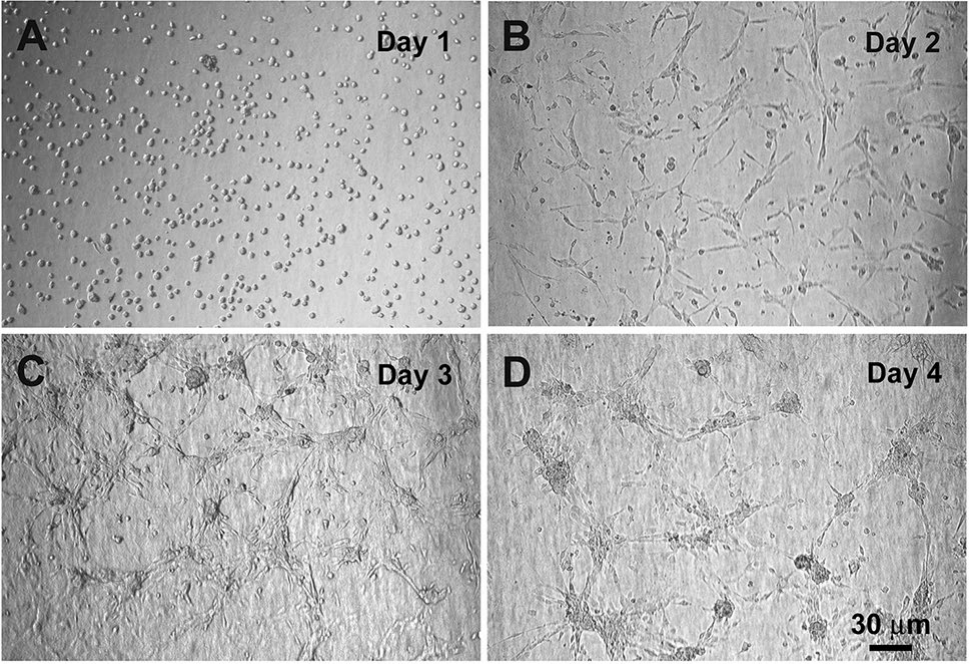
Growth of cultured spiral ligament fibrocytes from P10 mouse shown on sequential days after seeding onto collagen gels. **A**, day 1. **B**, day 2. **C**, day 3. **D**, day 4. The density of cells decreased daily, whilst the presence of processes extending from the cells increased. Images shown are from the same experiment and are representative of 5 experiments. Scale bar, 30 µm, applies to all the panels

Cells were seeded onto collagen I gels in order to allow them to grow in a 3D environment, as would occur in the spiral ligament. After initial seeding, the fibrocytes appeared rounded with no processes or connections. As time progressed, the cells became more interconnected and less flat than those cultured on 2-D collagen-coated surfaces (see Mahendrasingam et al. 2022). Cells were examined for morphological changes over time (*n* = 5 runs), Fig. 1 shows a representative example. Initially the cells appeared very round and isolated (Fig. 1A) before beginning to move around, spread out and make connections within an hour after seeding. On the second day, the cells appeared plumper, elongated, interconnected and fewer in number (Fig. 1B). This continued over the next 2 days with the cells becoming more connected and cell clusters or islands forming (Fig. 1C, D). This island-forming behaviour is similar to that found in the culture of guinea-pig lateral wall type III fibrocytes, where cells were incorporated within collagen gels (Kelly et al. 2012).

### Spiral Ligament Fibrocyte Marker Labelling

**Fig. 2:**
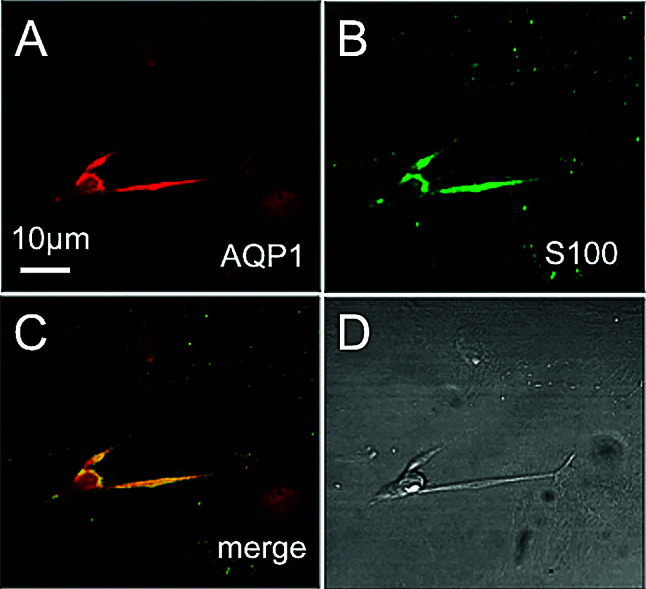
AQP1 and S-100 marker protein expression in a cluster of cultured fibrocytes on collagen I gel. **A**. Immunolabelling for the water channel, aquaporin (AQP1). **B**. Calcium binding protein, S-100. **C**. Merged image shows that these fibrocytes express both AQP1 and S-100. **D**. Bright field image. Images shown are from one experiment and are representative (*n* = 3). Scale bar = 10 µm and applies to all the panels

We tested for the presence of the water regulating membrane channel protein, aquaporin-1 (AQP1) and the cytoplasmic calcium binding protein, S-100 by immunolabelling (Fig. 2, representative images from 1 of 3 runs). These proteins are markers of fibrocyte types III (AQP1, Fig. 2A) and I, II and V (S-100, Fig. 2B), based on analysis of immunogold labelling in CD/1 mice (Mahendrasingam et al. 2011a). The merged image shows that both proteins are present in these cultured fibrocytes, with S-100 being more punctate in its expression (Fig. 2C). A bright field image of the cluster of fibrocytes is shown in Fig. 2D, revealing different morphologies. This result does not allow a conclusive identification of the type or types of fibrocyte within the culture, based on previous experiments in fixed mouse cochlear lateral wall (Mahendrasingam et al. 2011a). It could be that the principal fibrocyte in the culture is type III, but with additional expression of typically non-type III proteins as a consequence of growth in culture.

### Whole-Cell Voltage Clamp Experiments

**Fig. 3:**
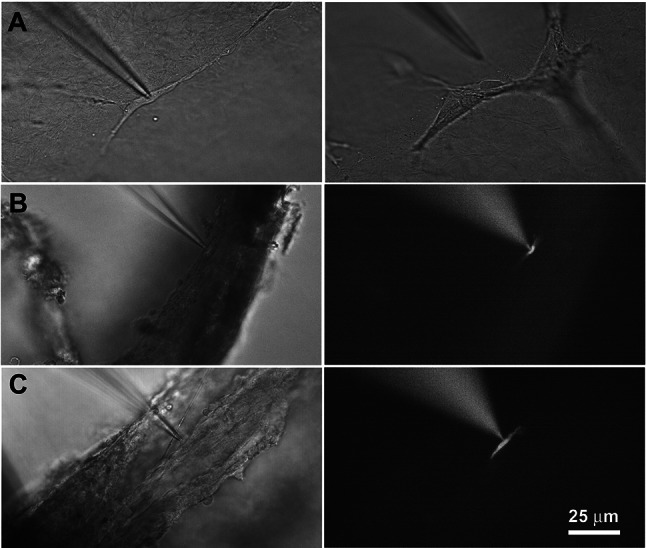
Fibrocyte images taken immediately before or during whole-cell recording, showing the three different preparations used. **A**. Fibrocytes cultured on gel surface. The patch pipette is shown just above the target cell (left) or further above a group of fibrocytes, and in line with the oval-shaped target cell (right). **B**. Cochlear slice preparation with patched fibrocyte in lateral wall originally adjacent to scala media (left), viewed with fluorescent illumination (right). The pipette was filled with fluorescein dye (dextran 3000) which dialyses the cell (a recording from this cell is shown in Fig. 4**B**). **C**. Dissected lateral wall preparation with patched fibrocyte (left), viewed with fluorescent illumination (right). Pipette filled with dextran 3000. The 25-µm scale bar applies to all the panels

Experiments were performed on fibrocytes cultured in collagen gels or ‘native’ fibrocytes within the cochlear lateral wall (spiral ligament), either from cochlear slices or excised portions of the spiral ligament. We refer to these groups as gel, slice and SL respectively, and examples are shown in Fig. 3. Examination of Fig. 3A gives an indication of the variety of morphological types encountered in the cell cultures and shows that some were clearly isolated, whereas others presented in clusters. In order to improve visualisation of voltage-clamped cells recorded in situ within the lateral wall (slice and SL), patch pipettes were filled with a fluorescent dextran dye which dialysed the cell (Fig. 3B, C). In some recordings, mainly from the cell cultures (gel), cell capacitance was measured. It was found to be bimodally distributed, with the majority of cells ranging from 5.0 to 19.5 pF (12.5 ± 4.4 pF, mean ± SD, *n* = 16), indicative of single, uncoupled cells, and a minority ranging from 27.5 to 35.4 pF (32.6 ± 3.6 pF, mean ± SD, *n* = 4, all gel), indicative of approximately 3 electrically coupled cells.

**Fig. 4:**
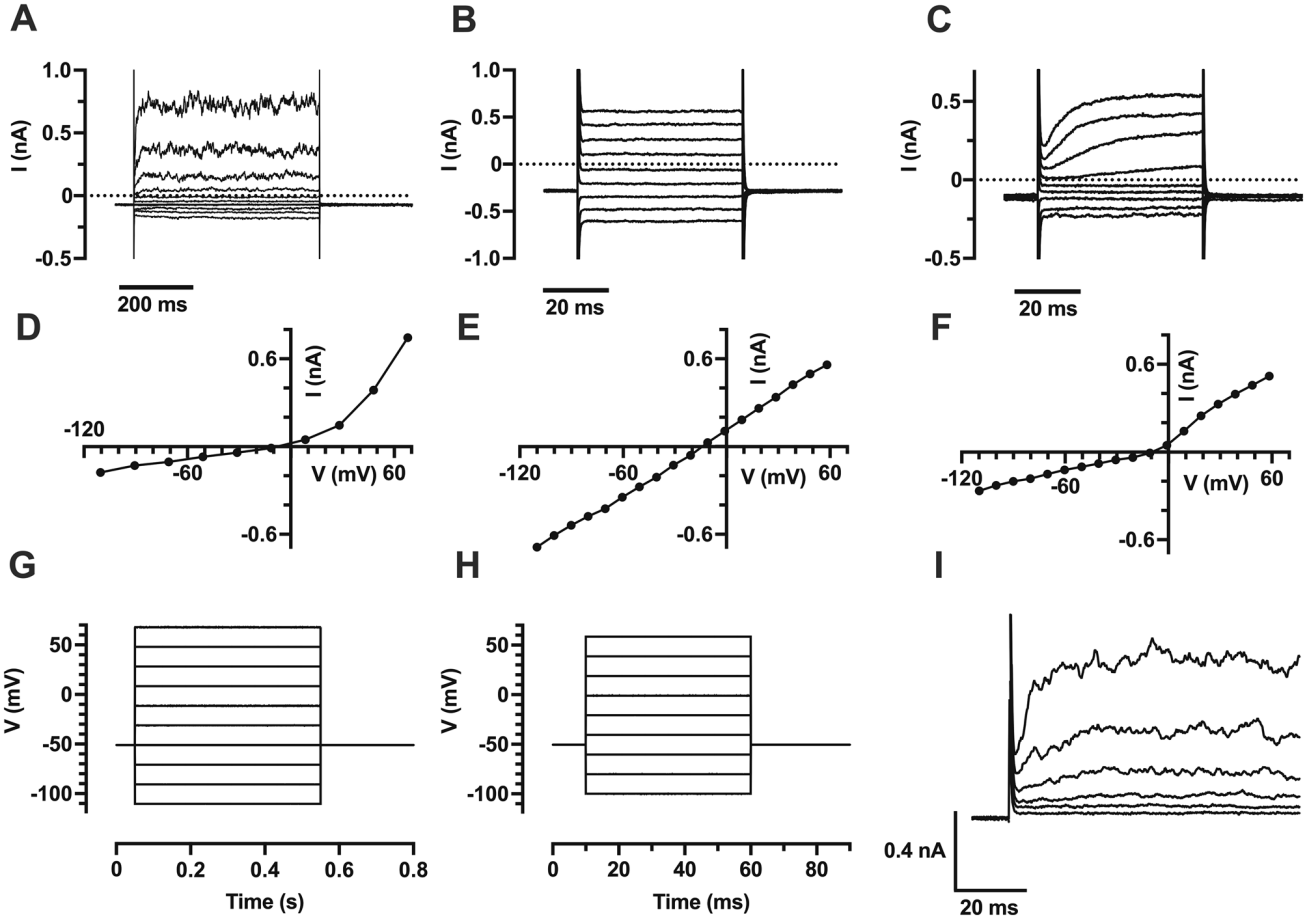
Examples of whole-cell voltage-clamp recordings from fibrocytes within the three preparations. **A**. Family of currents from fibrocyte on gel surface. **B**. Family of currents from fibrocyte in lateral wall, slice preparation. **C**. Family of currents from fibrocyte in dissected portion of lateral wall. **D**. I–V plot from cell shown in **A**. **E**. I–V plot from cell shown in **B**. **F**. I–V plot from cell shown in **C**. **G**. Voltage clamp steps for cell shown in **A**. **H**. Voltage clamp steps for cell shown in **B** and **C**, some steps removed for clarity. **I.** A subset of currents from the cell shown in **A** displayed on a faster time scale to permit comparison with **B** and **C**. Only currents in response to positive voltage steps are shown. Capacitative transients mark the beginning of the voltage step

When fibrocytes were held in voltage clamp at − 50 mV and stepped to different voltages above and below the holding potential, all the cells had a non-specific or ‘leak’-type conductance that was not time-dependent but was variable in size, and some cells had an outwardly rectifying current in addition, which activated with variable kinetics. Typical examples of recordings from the three preparations are shown in Fig. 4A, B and C, with their respective current–voltage (I–V) graphs (Fig. 4D–F) and the voltage steps (Fig. 4G, H). Comparing the three preparations, the proportions of cells showing increased conductance at depolarised potentials (outward rectification) were 44.4% in gels (4/9 cells, Fig. 4A, D), 0% in slices (0/5 cells, Fig. 4B, E) and 40.0% in SL (4/10 cells, Fig. 4C, F). To facilitate visual comparison of outward relaxations, some of the currents recorded from the cell shown in Fig. 4A, where longer voltage steps were used, are shown at a faster timescale (Fig. 4I). Generally speaking, in cells showing outward rectification, it was only seen at positive voltages (e.g. Fig. 4D, F). Currents activated rapidly at the most positive voltages and could be fitted with single exponential functions, with time constants ranging from 5 to 14 ms for the currents shown (Fig. 4C, I). The outwardly rectifying current also appeared noisy, at least in some cells (Fig. 4A), indicative of a large single channel conductance.

**Fig. 5:**
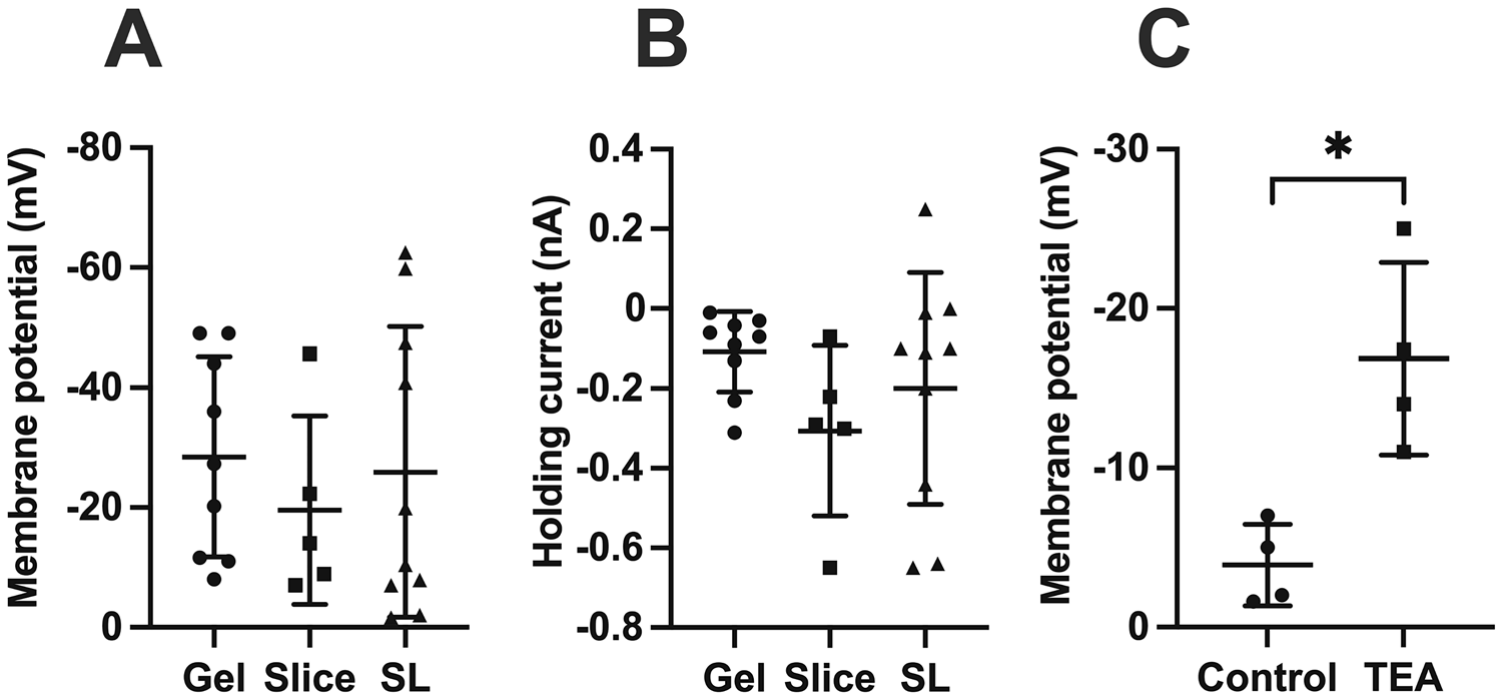
Comparison of membrane potential and holding current between fibrocytes in the three preparations (gel, slice, and SL (spiral ligament)). **A**. Membrane potential of fibrocytes, gel, slice and SL, *n* = 9, 5, 10 cells, respectively. ANOVA F (2,21) = 0.32, *p* = 0.73 (ns). **B**. Holding current measured at − 50 mV, *n* values as for **A**. ANOVA F (2,21) = 1.32, *p* = 0.29 (ns). **C**. Effect of TEA (10 mM) on membrane potential in four fibrocytes in gel and SL, *p* = 0.016 (paired *t*-test, *t* = 4.93, 2 tailed, 3 df). Each plot shows individual data points together with mean ± SD, significance (*p* < 0.05) indicated by asterisk

We examined the membrane potential of the cells in each group, together with the holding current at − 50 mV (Fig. 5A, B). Fibrocyte membrane potentials were (mean $\pm$ SD) − 28.5 $\pm$ 16.7 mV (gel, *n* = 9), − 19.6 ± 15.7 mV (slice, *n* = 5) and − 25.9 ± 24.3 mV (SL, *n* = 10). These values were not significantly different (ANOVA, F (2,21) = 0.32, *p* = 0.73, Fig. 5A). Holding currents measured at− 50 mV were also not significantly different (ANOVA, F (2,21) = 1.32, *p* = 0.29, Fig. 5B).

**Fig. 6:**
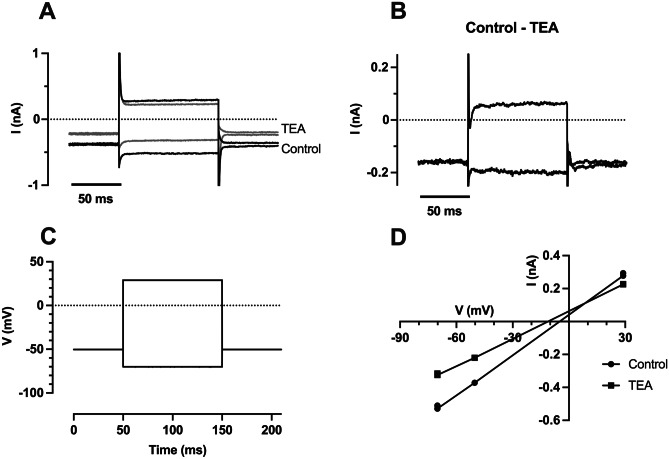
Effect of TEA on whole-cell currents in a fibrocyte cultured on gel surface. **A** Currents recorded in control and in the presence of TEA (10 mM). **B**. TEA-sensitive currents. The currents shown in A were averaged at each voltage (*n* = 2) and then subtracted (control − TEA). The large capacitative transients at the step onset and offset was a consequence of the individual transients differing slightly, and are shown truncated. **C**. Voltage clamp steps used in **A**. **D**. I–V plot of cell shown in **A** in the presence (squares) and absence of TEA (circles). The reversal potential of the TEA-sensitive current was + 10 mV in this experiment. The current acts to depolarise the resting membrane potential, as deduced from the shift in zero-current voltage

As some of the recordings showed outward rectification, indicating the presence of a voltage-dependent K^+^ channel, we tested whether the K^+^ channel blocker TEA would reduce the current. Starting with the cultured fibrocytes (*n* = 3), we observed a small but reversible reduction in current at the three voltages tested in the presence of locally applied TEA (10 mM, Fig. 6A, C). The TEA-sensitive current (Fig. 6B) reversed between + 10 mV and + 22 mV (Fig. 6D shows a reversal at + 10 mV), which is not consistent with a K^+^ channel block since the calculated K^+^ equilibrium potential was − 100 mV.

**Fig. 7:**
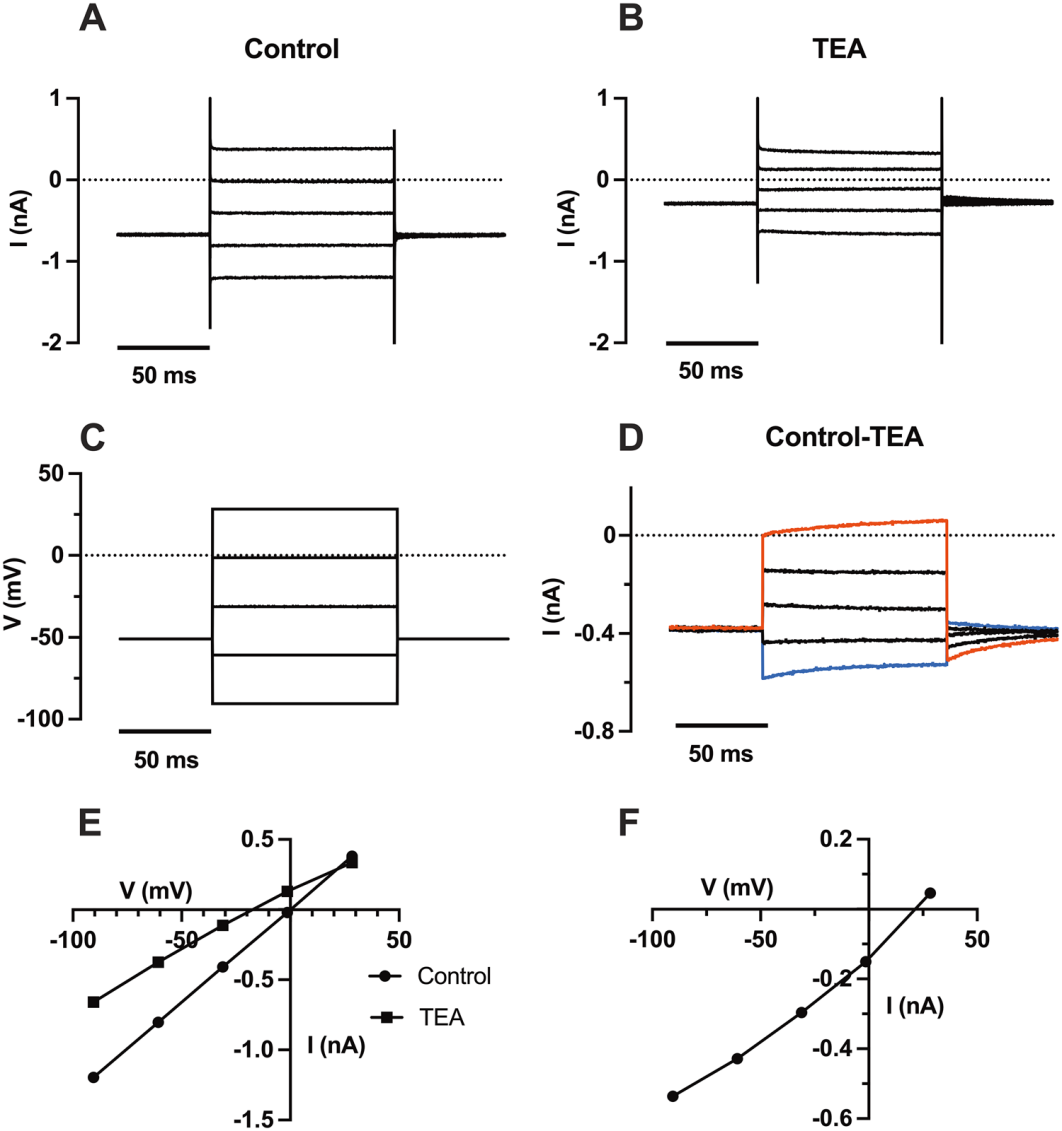
Effect of TEA on whole-cell currents in a fibrocyte recorded in an excised piece of spiral ligament (P8). **A**. Family of currents recorded under control conditions. **B**. Family of currents recorded in the presence of external TEA (10 mM). **C**. Voltage-clamp steps used in **A** and **B**. **D**. TEA-sensitive currents. The currents shown in **B** were subtracted from those shown in **A** (control − TEA). The current relaxations seen at the most positive and negative voltages (onsets and offsets) indicate an underlying voltage-dependence of the TEA block, positive voltages increasing the block. **E**. I–V plot of the currents recorded in control and in TEA. **F**. I–V plot of TEA-sensitive current, showing slight outward rectification. Current measurements were measured at the middle of the voltage steps, averaged over 20 ms. The reversal potential =  + 20 mV

We therefore investigated the TEA block in more detail, using a larger number of voltage steps in order to measure the reversal potential more accurately. These experiments were performed on the excised spiral ligament; an example is shown in Fig. 7. The currents recorded in the presence and absence of TEA (Fig. 7A, B) in response to a series of voltage steps (Fig. 7C), are shown together with the I–V curves (Fig. 7E). The positive reversal potential for the TEA-sensitive current (+ 25.6 ± 12.6 mV, mean $\pm$ SD, *n* = 3) is consistent with TEA blocking a non-specific cation current (with a calculated permeability ratio (P_*Na*_/P_*K*_) of 3.1 to account for the positive reversal potential). Also shown are the TEA-sensitive currents (Fig. 7D) and the corresponding I–V curve (Fig. 7F). The TEA-sensitive currents were obtained by subtracting currents in the presence of TEA from the controls. The current relaxations seen for the largest steps (-90 mV and +30 mV), and the tail currents produced on repolarisation, indicate a slow voltage-dependent block, increasing with depolarisation. The tail currents relaxed with a monoexponential time course with time constants of 30–40 ms for the larger tail currents where the best fits were obtained. In agreement with the voltage-dependence, the I–V curve of the TEA-sensitive current shows a slight outward rectification (Fig. 7F). As can be seen from the I–V plots in control and in TEA (Fig. 7E), TEA produced a negative shift in the zero-current potential (membrane potential). The membrane potential increased from − 3.9 ± 2.6 mV to − 16.9 ± 6.0 mV in the presence of TEA (*p* = 0.016, paired *t*-test, two-tailed, *t* = 4.93, df = 3, *n* = 4, Fig. 5C). Thus the TEA-sensitive current acts to depolarise the resting membrane potential.

## DISCUSSION

Our long-term goal is to assess whether cochlear fibrocytes cultured on the surface of collagen I gels might represent a suitable source of replacement fibrocytes for implantation in the cochlea lateral wall to replace those lost during age-related hearing loss. As an initial step towards this goal, we have assessed the electrical properties of cochlear fibrocytes in 3D cell culture and compared them with the electrical properties of native fibrocytes, in both slices and in excised spiral ligament. Additionally, we examined the expression of two key marker proteins in 3D-cultured cochlear fibrocytes (the water channel aquaporin, a type III fibrocyte marker, and the calcium-binding protein S-100, principally expressed in fibrocyte types I, II and V). The fact that cultured fibrocytes labelled for both markers has prevented us from a definitive identification of fibrocyte type (Mahendrasingam et al. 2011a). This might be related to the culture conditions or to the immature state of the fibrocytes (Mahendrasingam et al. 2022). One possibility is that they most closely resemble type III fibrocytes (since they label for aquaporin), with some upregulation of S-100 presumably as an adaptation to the absence of other fibrocyte types (Wu and Marcus 2003; Mahendrasingam et al. 2011a). A common observation was of a long thin bipolar morphology which would normally be typical of type I fibrocytes. Even so, type III fibrocytes in cell culture can also appear elongated, producing a bipolar-like morphology (Kelly et al. 2012). Thus, in our fibrocyte cell culture, it is not possible to arrive at a definitive conclusion about fibrocyte type.

A feature of our fibrocyte cultures was the formation of cell clusters or islands after around 2 days. This behaviour was previously reported in type III fibrocyte cell cultures from guinea-pig, where cells were grown within the collagen gel matrix and were shown to exert contractile forces on the gel (Kelly et al. 2012). A clear difference is that in our experiments cells were seeded on the surface of the gel to retain accessibility for the electrode, and not mixed within the gel. Thus the biophysical interactions between cell and gel are likely to be different. Nevertheless, the ability of the cells to clump and develop networks suggests some trophic factors are present attracting connectivity. As gap junction connections were found by Kelly et al. (2012), this suggests a similar process is occurring here and that the cells are attempting to form a syncytium.

In terms of their electrical properties, at least qualitatively we find a closer similarity between the 3D-cultured fibrocyte population to native fibrocytes from the dissected spiral ligament, rather than those recorded from cochlear slices. While we found no significant differences in membrane potential or holding current between the three preparations, we note that a lower proportion of fibrocytes from the slice group showed outward rectification in the I–V relation (0%) compared to the other two groups (40–44%). It could simply be that we need to improve the slicing procedure to achieve less disparity. Nonetheless, the similarity in electrical behaviour between the 3D-cultured fibrocytes and those within the excised spiral ligament does indicate that the culturing process does not greatly affect the membrane properties of the fibrocytes.

Our mean values for fibrocyte membrane potential ranged from − 20 mV to − 29 mV. While this is more negative than is typically found for native fibrocytes in microelectrode studies (0 −  + 7 mV, see “Introduction”), it is less negative than has been reported for other cultured fibrocytes (type I, stabilising at ~  − 80 mV, Shen et al. 2004). Our mean value for cell capacitance for the majority of cells, 12.5 pF, is suggestive of single cells rather than several cells electrically coupled together. In recordings from immature and thus electrically uncoupled fibrocytes in situ, capacitance values ranged from 3 to 20 pF, close to our values (Kelly et al. 2011). In cultured fibrocytes, we did occasionally find recordings with larger capacitance values and slower capacitative transients, as would be expected from electrically coupled cells, but these were excluded from further analysis. This was due to the inherent problems in successfully controlling membrane voltage and ionic gradients through a single patch pipette in electrically coupled cells. Even so, the apparent presence of some cell electrical coupling suggests that gap junctions can form between fibrocytes in 3D cell culture.

The finding that TEA, a broad-spectrum K^+^ channel blocker, blocks a non-specific or leak current in fibrocytes was unexpected. TEA has been found to block voltage-dependent non-specific cation currents before, in rat cortical neurons, at least at high concentration (35 mM: Alzheimer 1994). The fact that TEA blocks a component of what is usually described as a leak current does indicate that it flows through an ion channel, rather than through a parallel pathway, such as the seal between cell membrane and pipette, or through a damaged cell membrane. While we cannot at present identify the type of cation channel carrying the TEA-sensitive current, the evidence suggests a single channel type. If more than one channel type were responsible, a more complex time course would be expected, each channel exhibiting a different characteristic time course in response to the block, or to voltage changes. The temporal constancy of the current, albeit with a weak voltage-dependence, and the mono-exponential tail currents, are suggestive of a single channel type.

Given that the TEA-sensitive channel has a relatively high Na^+^ permeability which acts to depolarise the fibrocytes, the question arises as to its physiological significance. Previous studies of cochlear K^+^ transport, where a double-barrelled K^+^-sensitive microelectrode was advanced through the cochlear lateral wall towards scala media, have shown the presence of an electrical syncytium comprising type 1 fibrocytes and the lateral aspect of the stria vascularis, comprising basal and intermediate cells (Adachi et al. 2013; Yoshida et al. 2016). The membrane potentials of these cells were found to be small and positive (~ + 7 mV), suggestive of a significant Na^+^ permeability. In accord with this, substantially reducing perilymphatic Na^+^ concentration produced a hyperpolarisation of the fibrocyte (syncytium) membrane potential and a reduction in endocochlear potential (Yoshida et al. 2016). The TEA-sensitive cation conductance likely contributes to the fibrocytes Na^+^ permeability. The importance of this positive membrane potential is that it helps establish a significant electrochemical gradient in favour of K^+^ efflux from intermediate cells through their apical Kir 4.1 channels into the intrastrial space. This establishes the positive voltage (+ 85 mV) in the intrastrial space which in turn helps produce the positive endocochlear potential (Marcus et al. 2002; Nin et al. 2008).

Our results, although based on small samples, indicate that cochlear fibrocytes grown on collagen I gels have qualitatively similar membrane currents to native fibrocytes in the spiral ligament. This is a positive outcome when considering the potential of cultured fibrocytes to replace those lost during age-related hearing loss. The next major step is to determine whether these cultured fibrocytes can replace lost fibrocytes, initially in vitro before testing in vivo. Regarding the fibrocyte electrical properties, important questions remain as to the identity of the ion channels responsible for the ion currents described here, and how they shape the cells’ physiological function. It will be important to find out whether the cell culturing procedure does indeed produce a population of predominantly type III fibrocytes as we suggest, and to what extent fibrocyte electrical properties depend on their type and maturity.

## Data Availability

Data can be made available on request.
